# Liver X Receptor Alpha Is Important in Maintaining Blood-Brain Barrier Function

**DOI:** 10.3389/fimmu.2019.01811

**Published:** 2019-07-31

**Authors:** Elien Wouters, Nienke M. de Wit, Jasmine Vanmol, Susanne M. A. van der Pol, Bert van het Hof, Daniela Sommer, Melanie Loix, Dirk Geerts, Jan Ake Gustafsson, Knut R. Steffensen, Tim Vanmierlo, Jeroen F. J. Bogie, Jerome J. A. Hendriks, Helga E. de Vries

**Affiliations:** ^1^School of Life Sciences, Biomedical Research Institute, Hasselt University, Diepenbeek, Belgium; ^2^Department of Molecular Cell Biology and Immunology, Amsterdam Neuroscience, MS Center Amsterdam, Amsterdam UMC, Vrije Universiteit Amsterdam, Amsterdam, Netherlands; ^3^Department of Medical Biology, Amsterdam UMC, University of Amsterdam, Amsterdam, Netherlands; ^4^Center for Nuclear Receptors and Cell Signaling, University of Houston, Houston, TX, United States; ^5^Division of Clinical Chemistry, Department of Laboratory Medicine, Karolinska Institute, Stockholm, Sweden; ^6^Division Translational Neuroscience, School for Mental Health and Neuroscience, Maastricht University, Maastricht, Netherlands

**Keywords:** blood-brain barrier, permeability, endothelium, liver X receptors, neuroinflammation

## Abstract

Dysfunction of the blood-brain barrier (BBB) contributes significantly to the pathogenesis of several neuroinflammatory diseases, including multiple sclerosis (MS). Potential players that regulate BBB function are the liver X receptors (LXRs), which are ligand activated transcription factors comprising two isoforms, LXRα, and LXRβ. However, the role of LXRα and LXRβ in regulating BBB (dys)function during neuroinflammation remains unclear, as well as their individual involvement. Therefore, the goal of the present study is to unravel whether LXR isoforms have different roles in regulating BBB function under neuroinflammatory conditions. We demonstrate that LXRα, and not LXRβ, is essential to maintain barrier integrity *in vitro*. Specific knockout of LXRα in brain endothelial cells resulted in a more permeable barrier with reduced expression of tight junctions. Additionally, the observed dysfunction was accompanied by increased endothelial inflammation, as detected by enhanced expression of vascular cell adhesion molecule (VCAM-1) and increased transendothelial migration of monocytes toward inflammatory stimuli. To unravel the importance of LXRα in BBB function *in vivo*, we made use of the experimental autoimmune encephalomyelitis (EAE) MS mouse model. Induction of EAE in a constitutive LXRα knockout mouse and in an endothelial specific LXRα knockout mouse resulted in a more severe disease score in these animals. This was accompanied by higher numbers of infiltrating leukocytes, increased endothelial VCAM-1 expression, and decreased expression of the tight junction molecule claudin-5. Together, this study reveals that LXRα is indispensable for maintaining BBB integrity and its immune quiescence. Targeting the LXRα isoform may help in the development of novel therapeutic strategies to prevent BBB dysfunction, and thereby neuroinflammatory disorders.

## Introduction

Liver X receptors (LXRs) belong to a large family of nuclear receptors which upon activation stimulate gene transcription (1). Two LXR isoforms exist in mammals, termed LXRα (NR1H3) and LXRβ (NR1H2), which share over 75% amino acid sequence identity. In the nucleus LXRs form obligate heterodimers with the retinoid X receptor (RXR), together forming the LXR/RXR complex. LXRs play an important role in cholesterol and lipid metabolism. The best described process involving LXR function is reverse cholesterol transport where LXRs facilitate the elimination of excess cholesterol in response to cholesterol precursors or oxysterols (2). However, LXRs appear to be involved in a much broader spectrum of functions.

Recent studies identified LXRs as promising targets to interfere in inflammatory signaling pathways. LXR activation induces anti-inflammatory actions in macrophages by antagonizing NF-κB signaling (3). In the central nervous system (CNS), LXR agonists inhibit the production of proinflammatory cytokines and chemokines in stimulated microglia and reactive astrocytes (4). In several animal models of different CNS disorders, including stroke, Alzheimer's disease (AD), and multiple sclerosis (MS), the activation of LXRs results in a reduction of neuroinflammation, suggesting that LXR targeting may be effective in the treatment of neuroinflammatory disorders (5–8).

The main players in the neuroinflammatory process are proinflammatory cytokines and chemokines. These inflammatory mediators are produced locally within the CNS by glial cells or by leukocytes, which are recruited from the periphery following blood-brain barrier (BBB) breakdown (9). One of the pathological hallmarks of BBB dysfunction seen in neuroinflammatory disorders is increased permeability due to loss of tight junctions and increased leukocyte extravasation (10). During the extravasation process, chemokines presented by the inflamed brain endothelium guide the rolling and firm adhesion of leukocytes on the brain endothelial cell surface. Next, the interaction of integrins on leukocytes with brain endothelial cell adhesion molecules (CAMs) further induces their trans- or paracellular migration into the brain, illustrating the critical role of the BBB in mediating neuroinflammatory disorders (11, 12).

Brain endothelial cells tightly regulate BBB function and are regarded as the gatekeepers of the CNS (13, 14). So far, knowledge on the involvement of LXRs in BBB function is limited and is mostly linked to their function in cholesterol homeostasis. For instance, several studies indicate an upregulation of downstream ATP-binding cassette (ABC) cholesterol transporters after LXR agonism in primary brain endothelial cells (15, 16). Interestingly, LXR activation prevents the downregulation of the tight junctions occludin and zona occludens-1 in ischemic vessels in a mouse model of stroke, indicating that LXRs control BBB integrity (16). To date, it remains unclear whether LXRs regulate BBB function during a neuroinflammatory insult, and whether the LXRα and LXRβ isoforms have a distinct role in controlling BBB integrity. Therefore, the goal of the present study is to unravel whether LXR isoforms have different functions in regulating BBB function under neuroinflammatory conditions.

In this study, we show that LXRα, and not LXRβ, is essential to maintain BBB integrity. Impaired LXRα function in brain endothelial cells resulted in decreased barrier function and increased inflammation as marked by increased endothelial vascular cell adhesion molecule (VCAM-1) expression and enhanced trans-endothelial monocyte migration. Importantly, whole body knockout of LXRα and specific endothelial knockout of LXRα in a neuroinflammatory mouse model, resulted in enhanced extravasation of leukocytes into the brain together with increased VCAM-1 expression and reduced claudin-5 expression in the brain vasculature. Collectively, our findings show that LXRα is essential to maintain BBB function.

## Materials and Methods

### Cell Culture

The human immortalized cerebral microvascular endothelial cell line hCMEC/D3 (17) was grown in EGM-2 Endothelial Cell Growth Medium-2 BulletKit, including basal medium and supplement components according to the manufacturer's instructions (Lonza, Basel, Switzerland). All cell culture plates were coated with type I collagen (Invitrogen, Thermo Fisher Scientific, Leusden, The Netherlands). Cultures were grown to confluence at 37°C in 5% CO_2_. hCMEC/D3 cells were detached at 37°C with trypsin/EDTA in PBS (Gibco, Thermo Fisher Scientific).

### Lentiviral Short Hairpin RNA for LXRα and LXRβ Knockdown

Selective gene knockdown (KD) was obtained by using a vector-based short hairpin (sh) RNA technique as previously described (18). Recombinant lentiviruses were produced by co-transfecting subconfluent HEK 293T cells with the specific expression plasmids and packaging plasmids (pMDLg/pRRE, pRSV-Rev, and pMD2G) using calcium phosphate as a transfection reagent. HEK 293T cells were cultured in Dulbecco's modified eagle medium (DMEM) supplemented with 10% fetal calf serum (FCS) and 1% penicillin/streptomycin. Cells were cultured at 37°C in 5% CO_2_. Infectious lentiviruses were collected 48 h after transfection and stored at −80°C. The KD efficiency of all five constructs for each LXR isoform was tested, and the most effective construct used in subsequent experiments for LXRα (NR1H3) was TRC22237, encoding sequence GTGCAGGAGATAGTTGACTTT that target nucleotides 1,043–1,063 of the NM_005693.3 RefSeq. For LXRβ (NR1H2) the most effective construct was TRC275326, encoding sequence GAAGGCATCCACTATCGAGAT that target nucleotides 1,193–1,213 of the NM_007121.5 RefSeq. Subsequently, lentiviruses expressing LXRα- or LXRβ-specific shRNA were used to transduce hCMEC/D3 cells. Control cells were generated by transduction with lentivirus expressing non-targeting shRNA (SHC002, Sigma-Aldrich, St. Louis, MO). Forty-eight hours after infection of hCMEC/D3 cells with the shRNA-expressing lentiviruses, stable cell lines were selected by puromycin treatment (2 μg/ml). The expression knockdown efficiency was determined by quantitative real-time PCR (qRT-PCR).

### RNA Isolation and qRT-PCR

Recombinant hCMEC/D3 cell lines (1 × 10^6^ cells/ml) expressing either LXRα shRNA, LXRβ shRNA, or non-targeting shRNA were seeded in 24-well plates in growth medium. Upon confluency, cells were treated with DMSO (VWR, Leuven, Belgium) or with 5 ng/ml TNFα and 5 ng/ml IFNγ (Peprotech, London, UK) for 24 h. EAE animals were sacrificed on day 23 post-adoptive transfer or day 36 post-immunization. Spinal cords were isolated and snap frozen in liquid nitrogen. Total RNA from cultures and tissues was extracted using Qiazol (Qiagen, Venlo, The Netherlands) and the RNeasy mini kit (Qiagen), according to the manufacturer's instructions. RNA concentration and purity were determined with a NanoDrop spectrophotometer (Isogen Life Science, De Meern, The Netherlands). cDNA was synthesized using qScriptTM cDNA SuperMix (Quanta Biosciences, VWR), following manufacturer's guidelines. qRT-PCR was carried out using SYBR green master mix (Applied Biosystems, Waltham, MA) and a Step One Plus detection system (Applied Biosystems). Primers used for qRT-PCR are shown in Table S1. Relative quantitation of gene expression was accomplished using the comparative Ct method. Data were normalized to the most stable reference genes, as previously described (19).

### Flow Cytometry

For flow cytometric analysis of VCAM-1, hCMEC/D3 cells (1 × 10^6^ cells/ml) were seeded in 24-well plates. At confluency, cells were treated with DMSO as vehicle control or with 5 ng/ml TNFα and 5 ng/ml IFNγ for 24 h. hCMEC/D3 cells were detached from 24-well plates using 1 mg/ml collagenase type I (Sigma-Aldrich). Washed cells were incubated with mouse anti-human VCAM-1 (AbD Serotec, Kidlington, UK) for 30 min at 4°C. Binding was detected using secondary goat anti-mouse Alexa Fluor 488 (Molecular Probes, Eugene, OR). Omission of primary antibodies served as negative control. Fluorescence intensity was measured using a FACS Calibur flow cytometer (Becton, Dickinson and Company, Franklin Lakes, NJ).

### Electric Cell-Substrate Impedance Sensing (ECIS) Assay

The ECIS^TM^ Model 1600R (Applied BioPhysics, Troy, NY) was used to measure the barrier resistance (Rb) of confluent monolayers of hCMEC/D3 cells expressing non-targeting, LXRα or LXRβ shRNA. 100.000 cells were seeded onto each well of an 8W10+ ECIS array (Ibidi, München, Germany). The impedance Z [Ohm's law, potential (V)/current (I)] was measured at multiple frequencies in real-time. ECIS-Wounding was carried out with a current of 5,000 μA at 60 kHz for 20 s to measure proliferation rate of the different cell groups. All ECIS measurements were subjected to a mathematical model to calculate the component of resistance attributed to cell-cell interactions, called barrier resistance (Rb).

### BBB Permeability Assay

Recombinant hCMEC/D3 cells expressing non-targeting shRNA, LXRα shRNA or LXRβ shRNA were seeded at a concentration of 100.000 cells/cm^2^ onto the upper side of 0.4 μm pore-size collagen-coated Costar Transwell filters (Corning, Corning, NY) in growth medium. Paracellular permeability for FITC-dextran (70 kDa in growth medium, Sigma-Aldrich) from apical to basolateral direction was determined by collecting samples from the lower chambers after 4 h. The fluorescence intensity of the medium in the basolateral compartment was measured using a FLUOstar Galaxy microplate reader (BMG Labtechnologies, Offenburg, Germany, excitation 485 nm and emission 520 nm).

### Monocyte Migration

The migrating capacity of isolated monocytes across a monolayer of hCMEC/D3 cells was determined as previously described (20). Briefly, recombinant hCMEC/D3 cells were grown to confluence onto the upper side of 0.4 μm pore-size collagen-coated Costar Transwell filters (Corning) in growth medium and were subsequently exposed to either vehicle or TNFα (5 ng/ml) for 24 h. After washing, 100 μl of primary human monocytes (1 × 10^6^ cells/ml) was added to the upper chamber. The human blood monocytes were isolated from buffy coats of healthy donors (Sanquin, Blood Bank, Amsterdam, The Netherlands) by Ficoll gradient and anti-CD14 beads (21). Following 8 h of migration at 37°C and 5% CO_2_ in air, the transmigrated monocytes were harvested and quantified using anti-CD14 beads (Flow-Count^TM^ Fluorospheres, Beckman Coulter, Brea, CA) and subsequent FACScan flow cytometer analysis (Becton). The level of migration was calculated as the percentage of migrated monocytes to total monocytes within the field.

### Immunocytochemistry

Recombinant hCMEC/D3 cells expressing non-targeting shRNA, LXRα shRNA or LXRβ shRNA were grown to confluency in eight-well μ-slides (Ibidi). Cells were washed with ice-cold PBS and fixed in pre-cooled methanol for 10 min at −20°C. Fixed cells were washed and blocked with PBS containing 5% normal goat serum. Subsequently, cells were incubated overnight at 4°C with the primary antibody claudin-5 (Invitrogen). Next, cells were washed and incubated with the secondary antibody goat anti-mouse IgG Alexa Fluor 488 (Invitrogen).

### Mice

Wild-type C57BL/6JOlaHsd mice were purchased from Envigo (Venray, The Netherlands). LXRα^−/−^ and LXRα loxP/loxP mice on a C57BL/6 background were kindly provided by prof. dr. J.Å. Gustafsson (University of Houston, Houston, USA) (22). Cdh5(PAC)-creERT2 transgenic mice on a C57BL/6 background were kindly provided by prof. dr. Ralf H. Adams (Max Planck Institute, Münster, Germany) (23). All animal experiments were approved by the institutional animal care and use committee of Hasselt University (protocol numbers: 201422 201615 and 201617). The generation of endothelial-specific LXRα-inducible knockout mice was established by crossing LXRα loxP/loxP mice with Cdh5(PAC)-creERT2 transgenic mice to obtain Cdh5(PAC)-creERT2+LXRαLoxP/LoxP mice (LXRα^flox/flox^Cdh5-Cre^+/−^ mice). LXRα loxP/loxP and Cdh5(PAC)-creERT2 littermates were used as controls. Recombination was induced by injecting 10-week-old females intra-peritoneal with 100 μl tamoxifen [Sigma-Aldrich; 20 mg/ml in corn oil (Sigma-Aldrich)] for 5 consecutive days.

### Induction and Clinical Evaluation of EAE

At the age of 11 weeks, female C57BL/6 mice were actively immunized subcutaneously with 200 μg myelin oligodendrocyte glycoprotein peptide (MOG_35−55_) emulsified in 100 μl complete Freund's adjuvant containing 4 mg/ml Mycobacterium tuberculosis (EK-2110 kit; Hooke Laboratories, Massachusetts, USA). Directly after MOG_35−55_ immunization and after 24 h, mice were intraperitoneally injected with 100 ng (C57BL/6JOlaHsd donor mice adoptive T cell transfer) or 40 ng (endothelial specific knockout mice) pertussis toxin (EK-2110 kit; Hooke Laboratories) to induce a normal or a mild EAE, respectively. Both the control group and experimental group of one experiment received the same amount of PTX. Mice were weighed and clinically evaluated daily for neurological signs of the disease according to manufacturer's mouse EAE scoring guide: 0: no clinical symptoms; 0.5: distal tail paralysis; 1: tail paralysis; 2: mild paraparesis and ataxia; 2.5: moderate paraparesis; 3: complete paralysis of the hind legs; 4: paralysis to the diaphragm; 5: death by EAE.

### T Cell Adoptive Transfer

At day 9 post-immunization, inguinal lymph nodes were isolated from wild-type C57BL/6 donor mice. Next, T cells were collected and cultured at a concentration of 7 × 10^6^ cells/ml in stimulation medium (RPMI medium supplemented with 0.5% Penicillin-Streptomycin, 20 μM β-mercaptoethanol, 10% FCS, 1% Non-Essential Amino Acid, 1% sodium pyruvate and 20 ng/ml IL-23 (Bio-Legend, London, UK) containing 20 μg/ml MOG_35−55_. After 2 days of incubation, activated cells were intraperitoneally injected into LXRα^−/−^ acceptor mice or wild-type littermates at a density of 15 × 10^6^ cells/ml in PBS.

### Immunohistochemistry

Mice were sacrificed on day 23 post-adoptive transfer or day 36 post-immunization. Brains and spinals cords were isolated and snap frozen in optimal cutting temperature (OCT) compound. Material was sectioned using a Leica CM3050 S cryostat (Leica Microsystems, Wetzlar, Germany) to obtain 10 μm slices. Staining was performed on brain and spinal cord sections mounted on coated glass slides (Menzel Gläser Superfrost PLUS, Thermo Scientific, Braunschweig Germany). For colocalization studies, brain sections were air-dried, fixed in ice-cold methanol for 10 min at −20°C, and blocked for 30 min in 10% normal swine serum in PBS. Subsequently, sections were incubated overnight at 4°C with primary antibodies (claudin-5, VCAM-1 and rhodamine-lectin) as indicated in Table S2. Biotin labeled swine anti-rabbit (1:500, Dako, Agilent, Amstelveen, The Netherlands) followed by Alexa 488 labeled streptavidin (1:400, Molecular Probes) was used to detect claudin-5. Sections were incubated for 1 h with their specific secondary antibody. Finally, sections were stained with Hoechst (dilution 1:1000, Molecular Probes) to visualize cellular nuclei and mounted with Mowiol mounting medium. Representative images were taken using a Leica DM6000 microscope (20x objective, Leica Microsystems).

To study immune cell infiltration, spinal cord sections were air-dried and fixed in ice-cold acetone for 10 min at −20°C. Non-specific staining was blocked using Dako protein block (Agilent, Santa Clara, CA) for 30 min. Afterwards, sections were incubated overnight at 4°C with primary antibodies (CD3 and F4/80, Table S2). Secondary goat anti-rat IgG Alexa Fluor 555 (1:400, Thermo Scientific) was used to detect CD3 and F4/80. Representative images were taken using a Nikon eclipse 80i microscope (10× objective) and NIS Elements BR 3.10 software (Nikon, Tokyo, Japan).

### Quantitative Analysis

Image J version 1.52c (https://imagej.nih.gov/ij/index.html) was used for quantitative analysis of the expression of claudin-5 by recombinant hCMEC/D3 cells expressing non-targeting shRNA, LXRα shRNA, or LXRβ shRNA. For the quantification of the area fraction of the double fluorescent staining of claudin-5 and VCAM-1 overlapping with lectin in control mice, LXRα^−/−^ mice and LXRα^flox/flox^Cdh5-Cre^+/−^ mice, four pictures spanning the hippocampus were taken per animal. The amount of infiltrated immune cells, positive for CD3 and F4/80, was determined by quantitative analysis of six pictures per animal spanning the whole spinal cord.

### Statistical Analysis

Data were statistically analyzed using GraphPad Prism v6 (GraphPad Software, La Jolla, CA, USA) and are reported as mean ± standard error of the mean (SEM). D'Agostino-Pearson omnibus normality test was used to test normal distribution. One-way ANOVA (three groups) with Tukey's multiple comparison correction, two-way ANOVA (four groups) with Sidak's multiple comparison correction, or two-tailed unpaired student *T*-test (two groups) were used for normally distributed data sets. The Mann-Whitney (two groups) analysis was used for non-parametric data sets. No correction, i.e., Bonferroni for multiple statistical comparisons was performed. ^*^P < 0.05, ^**^P < 0.01, ^***^P < 0.001, and ^****^P < 0.0001.

## Results

### LXRα Is Important in Maintaining BBB Integrity

**Figure 1:**
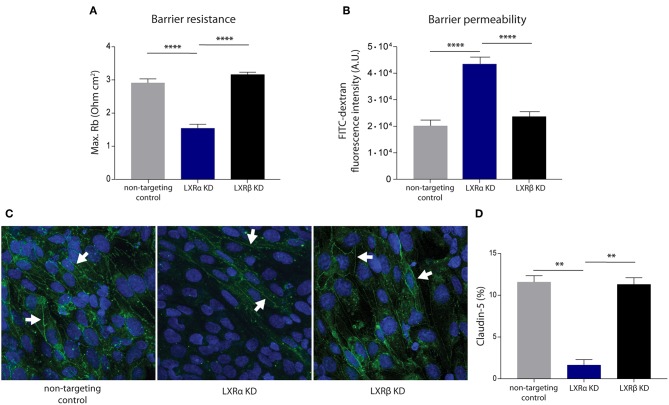
LXRα knockdown in the endothelial cell line hCMEC/D3 decreases BBB integrity. **(A)** LXRα knockdown cells show a significantly lower intercellular adhesion (Rb) compared to LXRβ knockdown and non-targeted control cells. Data are calculated from impedance measurements (Ohm cm^2^) ± SEM of two independent experiments performed in 4-fold. **(B)** Paracellular permeability of 70 kDa FITC-dextran was studied in time. LXRα knockdown cells were more permeable to FITC-dextran compared to control and LXRβ knockdown cells. Data are expressed as mean fluorescence intensity ± SEM after 4 h of three independent experiments performed in 3-fold. **(C)** Claudin-5 expression was studied using immunocytochemistry. Reduced claudin-5 expression (white arrows) was observed in LXRα knockdown cells compared to control and LXRβ knockdown cells (claudin-5; green, nuclei; blue). **(D)** Quantitative analysis of claudin-5 expression in non-targeting control, LXRα knockdown, and LXRβ knockdown cells. Statistical significance (one-way ANOVA, with Tukey's multiple comparison correction) is indicated with asterisks: ^**^p < 0.01, ^****^p < 0.0001.

To unravel the function of the LXRα and LXRβ isoforms in BBB integrity, we generated a brain endothelial cell line (hCMEC/D3) with a reduced expression of either LXRα or LXRβ. Transduction of brain endothelial cells with lentiviruses expressing LXRα- or LXRβ-specific shRNAs resulted in a reduced expression of LXRα or LXRβ, as determined by qRT-PCR. Only cells with at least 70% knockdown of expression were used in our study (Figure S1A). LXRα and LXRβ knockdown did not affect the proliferation rate of the endothelial cells (Figure S1B). ECIS analysis, used to measure transendothelial electrical resistance, was performed to determine the involvement of LXRα and LXRβ in brain endothelial cell barrier formation. The results of the one-way ANOVA showed a significant difference between the three cell types (*p* < 0.0001) of all in Figure 1 presented variables. Furthermore, *post-hoc* analysis revealed that knockdown of LXRα resulted in a significantly reduced barrier resistance compared to LXRβ knockdown cells (*p* < 0.0001) and the non-targeting control cells (*p* < 0.0001; Figure 1A). In accordance with a lower barrier resistance, the leakage of FITC-dextran was significantly (*p* < 0.0001) enhanced in LXRα deficient cells compared to cells lacking LXRβ and non-targeted control cells at 4 h (Figure 1B). Data on barrier formation or stability over the course of 4 h are not shown. Finally, immunocytochemical analysis revealed a decreased expression of the tight junction protein claudin-5 in LXRα-deficient cells (Figure 1C). In addition, quantification of the expression level of claudin-5 in the LXRα knockdown cells showed a significant decrease compared to LXRβ knockdown cells (*p* < 0.01) and non-targeting control cells (*p* < 0.01; Figure 1D), further strengthening the importance of LXRα in BBB function. Collectively, these findings demonstrate that LXRα, but not LXRβ, contributes to the formation of endothelial cell-to-cell junctions, thereby controlling BBB integrity.

### LXRα Knockdown Increases Monocyte Migration Across the BBB

During neuroinflammation, immune activation of the BBB facilitates the migration of leukocytes into the brain. To determine the involvement of LXRα and LXRβ in BBB function under neuroinflammatory conditions, we studied the expression levels of cytokines, chemokines, and adhesion molecules known to be involved in neuroinflammation. Although endothelial cells are capable of transrepression upon LXR activation (Figure S2A), without activation we found no isoform-specific increase or decrease in cytokine or chemokine expression under basal or inflammatory conditions (Figure S2B), using qRT-PCR.

**Figure 2:**
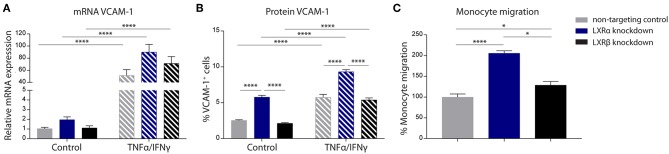
LXRα mediates immune activation of the endothelial cell line hCMEC/D3 and transendothelial migration of monocytes. **(A)** qRT-PCR of VCAM-1 mRNA expression and **(B)** FACS analysis of VCAM-1 protein expression levels in LXRα knockdown, LXRβ knockdown, and non-targeted control cells under basal and inflammatory conditions resulted in an increase of VCAM-1 in LXRα knockdown cells on protein level. Data are expressed as mean ± SEM of three independent experiments performed in 3-fold. **(C)** Primary monocyte migration across confluent monolayers of recombinant hCMEC/D3 cells expressing non-targeting shRNA, LXRα shRNA, or LXRβ shRNA. Knockdown of LXRα increased transmigration of monocytes across the barrier. Data are expressed as mean ± SEM of % migrated cells of total monocytes of two independent experiments performed in 4-fold. Statistical significance (**A**, **B**; two-way ANOVA, with Sidak's multiple comparison correction, and **C**; one-way ANOVA, with Tukey's multiple comparison correction) is indicated with asterisks: ^*^p < 0.05, ^****^p < 0.0001.

The results of the two-way ANOVA of VCAM-1 mRNA expression showed no main effect of cell type (*p* = 0.06). However, a significant main effect of inflammation (*p* < 0.0001) was present. In addition, there was no significant interaction effect between the cell type and inflammation (*p* = 0.08; Figure 2A). However, the results of the two-way ANOVA of the protein expression of VCAM-1 showed a significant main effect of cell type (*p* < 0.0001) and a significant main effect of inflammation (*p* < 0.0001), yet there was no significant interaction effect between the two (*p* = 0.83). *Post-hoc* analysis revealed that VCAM-1 protein expression levels were significantly increased in LXRα knockdown cells under basal (*p* < 0.0001) as well as inflammatory conditions (*p* < 0.0001) compared to LXRβ knockdown and non-targeted control cells (Figure 2B).

The one-way ANOVA revealed a significant difference (*p* < 0.0001) between the three cell types regarding monocyte migration. Consistent with increased VCAM-1 expression, endothelial LXRα knockdown resulted in a significantly increased migration of primary human monocytes across the endothelial barrier compared to LXRβ knockdown (*p* < 0.05) and non-targeting control (*p* < 0.0001; Figure 2C). Taken together, these results show that LXRα knockdown increases VCAM-1 expression on brain endothelial cells, stimulating transmigration of monocytes across the BBB.

### LXRα^−/−^ Worsens the Disease Score and Impairs BBB Function in a Mouse Model of Neuroinflammation

**Figure 3:**
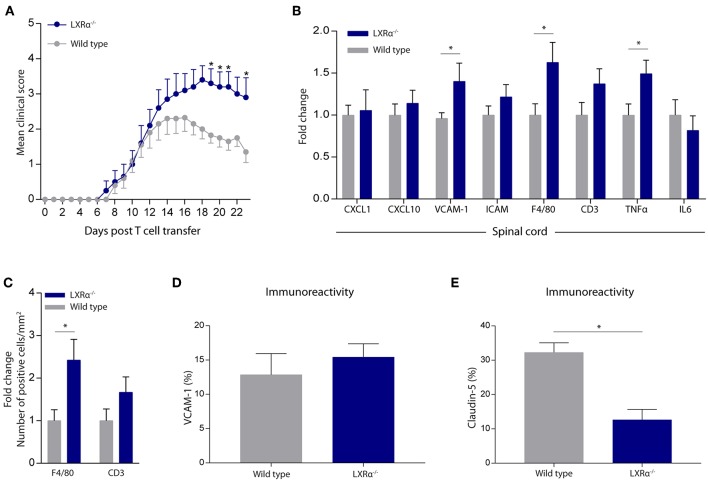
LXRα^−/−^ exacerbates the disease score in a neuroinflammatory mouse model. **(A)** Adoptive transfer EAE was induced in wild type (WT) and LXRα^−/−^ acceptor mice by immunization with MOG_35−55_ activated T-cells from WT donor mice. LXRα^−/−^ mice showed a higher mean clinical score during EAE compared to WT mice (*n* = 10 per group). **(B)** qRT-PCR analysis of neuroinflammatory marker expression in the spinal cord of WT (*n* = 7) and LXRα^−/−^ mice (*n* = 9). **(C)** Quantitative analysis of the number of infiltrated macrophages (F4/80) and T cells (CD3) into the spinal cord of WT (*n* = 8) and LXRα^−/−^ mice (*n* = 7). **(D)** Quantitative analysis of the immunoreactive area for VCAM-1 and **(E)** claudin-5 in WT (*n* = 5) and LXRα^−/−^ mice (*n* = 5). The values represent the mean ± S.E.M. Statistical significance (**A**; two-way repeated measures ANOVA, with Sidak's multiple comparison correction, **B**–**E**; Mann-Whitney *U*-test) is indicated with asterisks: ^*^p < 0.05.

Given the importance of LXRα in maintaining a functional BBB *in vitro*, we next sought to determine whether LXRα is involved in BBB function during neuroinflammation *in vivo*. For this purpose, we made use of the experimental autoimmune encephalomyelitis (EAE) MS mouse model. Because LXRs impact T cell proliferation and differentiation (5, 24, 25), we chose a T cell adoptive transfer model in which wild-type T cells are transferred to whole-body LXRα^−/−^ mice. A two-way repeated measures ANOVA showed a significant main effect of the two groups of mice (*p* < 0.0001), a significant main effect of time (*p* < 0.0001), and most importantly a significant interaction effect (*p* < 0.0001). Daily evaluation of the disease severity demonstrated an increase in EAE score in LXRα^−/−^ mice compared to wild-type mice. No differences were observed in disease onset (Figure 3A). The increase in mean clinical score was accompanied by increased inflammatory cytokine and chemokine mRNA expression in the spinal cord of LXRα-deficient animals. In line with our *in vitro* findings, lack of LXRα resulted in increased VCAM-1 mRNA expression (Figure 3B). Similar, LXRα deficiency increased the mRNA expression of F4/80, suggesting elevated infiltration of peripheral myeloid cells. Immunohistochemical analysis of the spinal cord confirmed the increased infiltration of macrophages (*p* < 0.05; Figure 3C). In the brain, no significant difference in the expression of VCAM-1 by endothelial cells was observed (Figure 3D). However, LXRα^−/−^ mice did show a significant decrease in claudin-5 expression (*p* < 0.05) compared to wild-type mice (Figure 3E). Collectively, these findings show that LXRα has a protective function during neuroinflammation.

### Endothelial Specific LXRα^−/−^ Aggravates Disease Progression in a Mouse Model of Neuroinflammation

To elucidate whether increased CNS infiltration of inflammatory cells in LXRα^−/−^ EAE mice is due to the absence of LXRα in the endothelium of the BBB, endothelial-specific knockouts of LXRα were generated by crossing LXRα loxP/loxP and Cdh5(PAC)-creERT2 transgenic mice (LXRα^flox/flox^Cdh5-Cre^+/−^). No significant difference (repeated measures two-way ANOVA, Sidak's multiple comparison correction) was observed in disease score between LXRα loxP/loxP and Cdh5(PAC)-creERT2 transgenic mice over time (data not shown). Therefore, both control groups were combined for further analyses.

**Figure 4:**
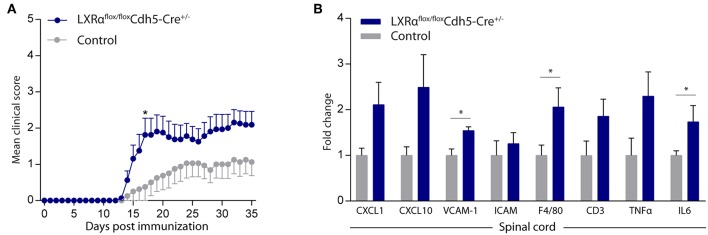
Endothelial specific knockout of LXRα worsens disease progression in a neuroinflammatory mouse model. **(A)** Mild EAE (mean clinical score 1–2 in control animals) was initiated in a cohort of 11-week-old Cdh5(PAC)-creERT2+LXRαLoxP/LoxP mice (LXRα^flox/flox^Cdh5-Cre^+/−^). LXRα loxP/loxP and Cdh5(PAC)-creERT2 transgenic mice were used as controls. LXRα^flox/flox^Cdh5-Cre^+/−^mice show a higher clinical score during the disease course compared with control mice (*n* = 8 per group). **(B)** qRT-PCR analysis of neuroinflammatory marker expression in the spinal cord of control (*n* = 8) and LXRα^flox/flox^Cdh5-Cre^+/−^ mice (*n* = 8). The values represent the mean ± S.E.M. Statistical significance (**A**; two-way repeated measures ANOVA, with Sidak's multiple comparison correction, **B**; Mann-Whitney *U*-test) is indicated with asterisks: ^*^p < 0.05.

**Figure 5:**
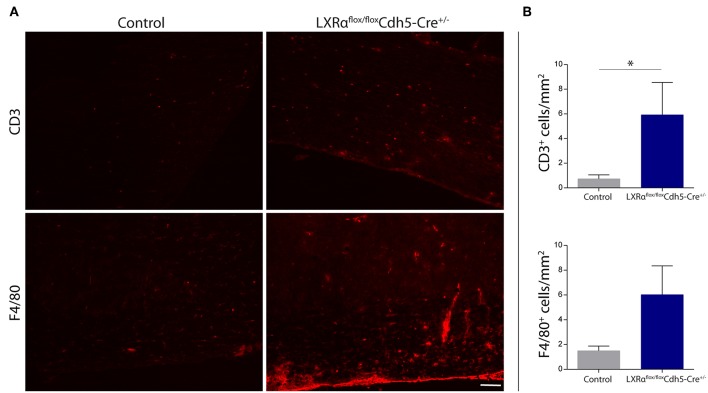
Increased leukocyte infiltration in the spinal cord of LXRα^flox/flox^Cdh5-Cre^+/−^ mice. **(A)** Immunofluorescent labeling of leukocytes (CD3, upper panel) or macrophages (F4/80, lower panel) in the spinal cord of control and LXRα^flox/flox^Cdh5-Cre^+/−^ mice. Bar: 100 μm. **(B)** Quantitative analysis of the number of infiltrated macrophages (F4/80) and T cells (CD3) into the spinal cord of control (*n* = 8) and LXRα^flox/flox^Cdh5-Cre^+/−^ mice (*n* = 8 per group). The values represent the mean ± S.E.M. Statistical significance (Mann-Whitney *U*-test) is indicated with asterisks: ^*^p < 0.05.

**Figure 6:**
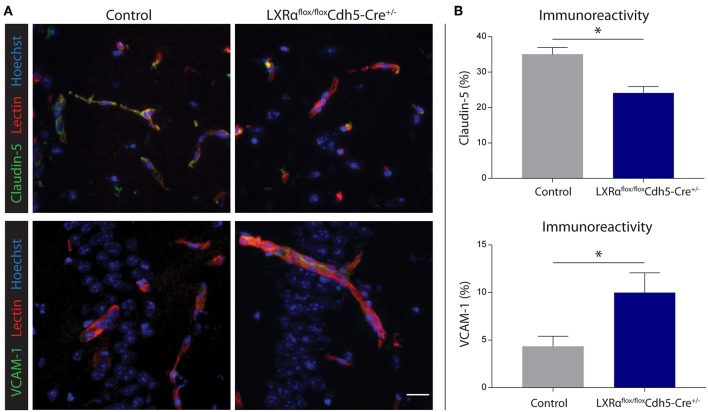
Endothelial specific knockout of LXRα results in decreased barrier integrity. **(A)** Immunofluorescent double labeling indicated that endothelial cells (red) of LXRα^flox/flox^Cdh5-Cre^+/−^ mice express less claudin-5 (green, upper panel) and more VCAM-1 (green, lower panel) compared to control animals. Nuclei were counterstained with Hoechst (blue). Bar: 20 μm. **(B)** Quantitative analysis of the immunoreactive area for claudin-5 and VCAM-1 in control (*n* = 5) and LXRα^flox/flox^Cdh5-Cre^+/−^ mice (*n* = 7). The values represent the mean ± S.E.M. Statistical significance (Mann-Whitney *U*-test) is indicated with asterisks: ^*^p < 0.05.

Endothelial knockout of LXRα resulted in a significant reduction in LXRα expression in endothelial cell isolates (Supplementary Materials and Methods, Figure S3). A two-way repeated measures ANOVA showed a significant main effect of the two groups of mice (*p* < 0.0001), a significant main effect of time (*p* < 0.0001), and most importantly a significant interaction effect (*p* < 0.0001). During a mild EAE (mean clinical score 1–2 in control animals), endothelial-specific deletion of LXRα resulted in a more severe mean clinical score compared to control animals with no difference in disease onset (Figure 4A). This increase in mean clinical score was associated with increased cytokine and chemokine expression in the spinal cord of endothelial-specific LXRα-deficient animals (Figure 4B). In addition, a significant increase in VCAM-1 mRNA expression together with enhanced F4/80 macrophage marker mRNA expression was found (*p* < 0.05; Figure 4B). Immunohistochemical analysis of the spinal cord showed a significantly increased migration of peripheral leukocytes (CD3, *p* < 0.05; Figures 5A,B). Moreover, immunohistochemical analysis of the brain tissue showed an enhanced expression of VCAM-1 (*p* < 0.05) and a decrease in claudin-5 expression (*p* < 0.05) in LXRα^−/−^ mice (Figures 6A,B). The expression levels of VCAM-1 were similar to the expression levels of VCAM-1 in the whole-body LXRα^−/−^ mice (Figure 3D vs. Figure 6B). Comparing claudin-5 expression levels between whole-body and endothelial specific LXRα^−/−^ mice revealed a significant higher expression of claudin-5 (*p* < 0.01) in the latter group (Figure 3E vs. Figure 6B). These results demonstrate that LXRα deficiency in endothelial cells aggravates the disease course in a mouse model of neuroinflammation.

## Discussion

The BBB is a highly specialized structure essential for CNS homeostasis. In this study, we determined the effect of both LXR isoforms on BBB integrity during neuroinflammation. Our experiments performed *in vitro* show that mainly LXRα, and not LXRβ, is important in maintaining proper barrier function. In addition, under neuroinflammatory conditions LXRα knockdown resulted in increased VCAM-1 expression by the endothelial cells, which was accompanied by an increase in monocyte migration across the barrier. Moreover, our *in vitro* findings were confirmed *in vivo* where endothelial specific knockout mice under neuroinflammatory conditions showed a higher disease score, increased peripheral leukocytes extravasation into the spinal cord, together with higher VCAM-1 expression and lower claudin-5 expression in the brain compared to control mice.

Our results demonstrate a difference in function between the LXRα and LXRβ isoform in brain endothelial cells. Even though LXRα is present in lower levels than LXRβ in the endothelial cells in mice and in the hCMEC/D3 cell line used in our experiments, we still observe a significant effect after knockdown of LXRα on BBB integrity, indicating that LXRα is essential in the regulation of BBB function. The two LXR isoforms share high sequence homology, but differ in their tissue distribution and function. LXRα is mainly expressed in the liver, intestine, adipose tissue, and macrophages and regulates for example reverse cholesterol transport in human macrophages and bile acid metabolism in the liver. On the other hand, LXRβ is more ubiquitously expressed and is involved in processes like lipid metabolism in the CNS and water transport in the pancreas (26, 27). A large amount of research has been performed using general LXR agonists that can target both LXR isoforms, thereby neglecting the possibility that both isoforms might exert different functions (16, 28, 29). Our results indicate that it is crucial to study the individual role of the distinct isoforms in different tissues, either by developing specific agonists or by generating a specific knockout in the tissue of interest.

We further show that whole-body and endothelium specific knockout of LXRα results in a decreased barrier integrity and increased inflammatory burden in a mouse model of neuroinflammation. Although the expression levels of VCAM-1 are similar between the two groups, comparing claudin-5 expression levels revealed a significant difference, where the endothelial specific LXRα^−/−^ mice show higher expression. This difference in expression level might be the result of the interaction between astrocytes and pericytes with the endothelial cells. Both cell types are important in maintaining BBB properties, and lacking LXRα might influence the functional interaction. Other studies have used synthetic agonists to investigate LXR function in the vasculature. For instance, the treatment of human umbilical vein endothelial cells (HUVECs) with GW3965, a LXR agonist, inhibited the adhesion of monocytes to endothelial cells (28). Furthermore, LXR activation by T0901317 in a mouse model of ischemic stroke selectively prevented the downregulation of occludin and ZO-1 on ischemic microvessels (16). A more recent paper demonstrated that LXR activation by GW3965 also positively modulated the microvasculature in an Alzheimer mouse model (29). However, these studies did not make a distinction between LXRα and LXRβ. Our results suggest that these beneficial processes at the endothelial level are controlled by the LXRα isoform, and not by LXRβ.

Several other studies showed that LXR activation is able to suppress EAE and reduce CNS inflammation (5, 24, 25). In these papers, the protective impact of the LXR agonists was attributed to their impact on T cell proliferation and differentiation. However, their effect on BBB function has not been described. The results of our study suggest that specifically the activation of LXRα might ameliorate the EAE disease course via regulating BBB integrity and inflammation. By maintaining BBB integrity, i.e., less VCAM-1 expression and maintaining tight junction expression, less immune cells might be able to infiltrate the brain. Interestingly, we only observed an effect on endothelial specific LXRα knockdown during a mild EAE (mean clinical score 1–2 in control animals), and not during a normal EAE (clinical score above 2—data not shown). This could partly be explained by the effect of pertussis toxin (PTX) on BBB permeability and leukocyte recruitment (30, 31). Therefore, by inducing a mild EAE using lower PTX concentration, BBB integrity changes caused by LXRα knockdown could still contribute to disease severity. Of note, Cdh5 is also expressed in some cells of the hematopoietic system, including macrophages (32), which implies that macrophages in our generated LXRα^flox/flox^Cdh5-Cre^+/−^ mice may also lack LXRα expression, which may influence disease to a certain extent. However, since we observe similar effects using our *in vitro* assays using brain endothelial cells that lack LXRα, we are confident that the majority of observed effects are due to the role of LXRα in the endothelium. It will be very interesting to define the impact of LXR isoform-specific agonists on BBB integrity and inflammation, once these become available. Nevertheless, it is important to take into account that hepatic LXRα activation promotes hepatic steatosis and dyslipidemia (33, 34). Therefore, targeting of LXRα via liposomes or adeno-associated viruses specific for endothelial cells would be useful.

So far, we can only speculate about the underlying pathways. One possible mechanism that could contribute to the observed effects, is ABCA1 induction by LXRs. This transporter is not only important for the efflux of intracellular free cholesterol, but also has an anti-inflammatory effect in both the brain and in the peripheral circulation (35–37). Moreover, ABCA1 is able to suppress metalloproteinase-9 (MMP9) expression in the ischemic brain (38). MMP9 is an important inducer of BBB damage presumably via the degradation of tight junction proteins and basement membrane extracellular matrix proteins (39). In macrophages, the stimulation of LXRs results in decreased MMP9 expression (40). Consequently, the induction of MMP9 expression in LXRα deficient endothelial cells could result in BBB damage. Interestingly, our LXRα knockdown cells showed higher MMP9 mRNA expression (data not shown). However, further studies are needed to determine whether this pathway is responsible for the LXRα mediated effects in endothelial cells.

Another possible mechanism is a process called epithelial to mesenchymal transition (EMT), which is driven by the transcription factor Snail. During EMT, the epithelial phenotype shifts through changes in gene expression, loss of cell polarity and cell-cell adhesion, and reorganization of the cytoskeleton, ultimately leading to a more migratory and invasive phenotype (41). Interestingly, in different cancer cell lines the presence or overexpression of LXRα positively contributes to their migratory abilities and Snail expression, whereas the opposite is observed in epithelial cells, where absence of LXRα results in a higher Snail expression (42–44). EMT has also been described for (brain) endothelial cells (EndoMT) and might underlie the observed changes in the endothelial cells when LXRα is absent (45–48).

In conclusion, we show that LXRs have different roles in regulating BBB function under neuroinflammatory conditions. More specially, we demonstrate that LXRα, and not LXRβ, is needed to maintain barrier integrity. Endothelial specific knockdown of LXRα *in vitro* and *in vivo* resulted in a more permeable barrier with less tight junctions, increased expression of adhesion molecule VCAM-1, and in an increased transendothelial migration of peripheral leukocytes across the barrier. Understanding the mechanisms by which BBB permeability is regulated during neuroinflammation may help in the development of therapeutic strategies, i.e., targeted delivery or selective activation of LXRα, to prevent BBB leakage and peripheral leukocyte infiltration during the early stages of neuroinflammatory diseases.

## Ethics Statement

This study was carried out in accordance with the recommendations of the institutional animal care and use committee of Hasselt University. The protocol was approved by the institutional animal care and use committee of Hasselt University (protocol numbers: 201422, 201615, and 201617).

## Author Contributions

EW, NdW, and JV are responsible for the generation of all data. SvdP, BvhH, DS, and ML gave technical support during experiments. EW and NdW wrote the manuscript. DG provided constructs for the generation of LXR knockdown brain endothelial cells. JG and KS provided the animals. JB and TV performed the adoptive T cell transfer and supervised the research. JH and HdV helped in designing the work and provided feedback on the manuscript. All the authors have read and approved the manuscript.

### Conflict of Interest Statement

The authors declare that the research was conducted in the absence of any commercial or financial relationships that could be construed as a potential conflict of interest.
