# Supplementary material for: Liver X Receptor Alpha Is Important in Maintaining Blood-Brain Barrier Function
Source: Front Immunol. 2019 Jul 31;10:1811. doi: 10.3389/fimmu.2019.01811 (PMC6685401; doi:10.3389/fimmu.2019.01811)
Supplement: Supplementary file 1 [file Table_1.DOCX]

**Supplementary materials and methods**

**Mouse brain capillary endothelial cell isolation**

Following tamoxifen injections, brains were isolated from 11 to 14 week old LXR𝛼^flox/flox^Cdh5-Cre^+/-^ and control mice. Meninges were removed by rolling the brain over sterile Whatman filter paper. Tissue was homogenized in isolation medium (HBSS, 10 mM Hepes and 0.1% BSA) using 10 strokes with a loose-fit dounce tissue grinder followed by 10 strokes with a tight-fit dounce tissue grinder (Sigma Aldrich). The homogenate was centrifuged (700*g* for 10 min at 4°C), and the resulting pellet was dissolved in 15% dextran solution (Sigma-Aldrich) in isolation medium. After a next centrifugation step (3000*g* for 25 min at 4°C), the pellet containing microvessels was digested in collagenase/dispase (2 mg/ml, Sigma-Aldrich) containing 10 µg/ml DNase (Sigma Aldrich) in culture medium (DMEM, 20% FCS, 1% amino acids, 2% sodium pyruvate, 50 μg/ml gentamycin) and incubated for 30 minutes at 37°C. Isolated brain capillary endothelial cells were washed in isolation medium and lysed in RIPA-buffer (150 mM sodium chloride, 1.0% Triton X-100, 0.5% sodium deoxycholate, 0.1% SDS, 50 mM Tris, pH 8.0) containing protease and phosphatase inhibitors (Roche, Mannheim, Germany).

**Immunoblotting**

Proteins were separated by SDS-PAGE and wet transferred to a PVDF membrane (GE Healthcare, Buckinghamshire, UK). Non-specific binding was blocked by incubating the membranes in 5% BSA in Tris-buffered saline containing 0.1% Tween-20 (TBS-T) for 1 hour. Afterwards, membranes were incubating with primary antibodies, LXRα (1:500, Abcam, Cambridge, UK) or β-actin (1:10000, Santa Cruz Biotechnology, Heidelberg,Germany) in 5% BSA in TBS-T overnight at 4°C. After washing in TBS-T, membranes were incubated for 1 hour at room temperature with a horseradish peroxidase-conjugated goat anti-mouse antibody (1:2000 for LXRα and 1:5000 for β-actin, Agilent) in TBS-T. Protein bands were detected using ECL western blot detection reagents (Thermo Fisher Scientific) and an ImageQuant LAS 4000 mini analyzer (GE Healthcare). For re-probing, membranes were stripped using a mild stripping buffer (0.2 M glycine, 0.1% SDS, 1% Tween-20, pH 2.2), followed by incubation in blocking buffer and primary antibody. Protein bands were quantified using ImageQuant TL 8.1 image analysis software (GE Healthcare).
