# Supplementary material for: Liver X Receptor Alpha Is Important in Maintaining Blood-Brain Barrier Function
Source: Front Immunol. 2019 Jul 31;10:1811. doi: 10.3389/fimmu.2019.01811 (PMC6685401; doi:10.3389/fimmu.2019.01811)
Supplement: Supplementary file 2 [file Table_2.DOCX]

***Table S1.*** Primers used for RT-qPCR

| **Gene** | **Forward primer** | **Reverse primer** |
| --- | --- | --- |
| h-CXCL2 | CGCCCCTGGCCACTGAACTG | TGCCCATTCTTGAGTGTGGCTATGA |
| h-CCL2 | TCAGCCAGATGCAATCAATG | TGGAATCCTGAACCCACTTC |
| h-CYCA | AGACTGAGTGGTTGGATGGC | TCGAGTTGTCCACAGTCAGC |
| h-GAPDH | CCATGTTCGTCATGGGTGTG | GGTGCTAAGCAGTTGGTGGTG |
| h-ICAM-1 | AGCTTCGTGTCCTGTATGGC | ACAGTCACTGATTCCCCGAT |
| h-IL-6 | GAGGAGACTTGCCTGGTGAA | GCTCTGGCTTGTTCCTCACT |
| h-LXR𝛼 | TGTCGGCTTCGCAAATG | CAAGGATGTGGCATGAGC |
| h-LXR𝛽 | CAAGTGCCTGGTTTCCTG | ACTCTGTCTCGTGGTTGTAG |
| h-VCAM-1 | AATGTTGCCCCCAGAGATACAACCG | GAGCTGCCTGCTCCACAGGA |
| h-YWHAZ | CTTGACATTGTGGACATCGG | TATTTGTGGGACAGCATGGA |
| m-CD3 | AACACGTACTTGTACCTGAAAGCTC | GATGATTATGGCTACTGCTGTCA |
| m-CXCL1 | GCCTATCGCCAATGAGCTG | CTGAACCAAGGGAGCTTCAGG |
| m-CXCL10 | GTGCCCACGTGTTGAGATCA | TGGTCTTAGATTCCGGATTCAGA |
| m-CYCA | GCGTCTCCTTCGAGCTGTT | AAGTCACCACCCTGGCA |
| m-F4/80 | GGATGTACAGATGGGGGATG | TCTGTGGTGTCAGTGCAGGT |
| m-TNFα | CCAGACCCTCACACTCAG | CACTTGGTGGTTTGCTACGAC |
| m-IL6 | TGTCTATACCACTTCACAAGTCGGAG | GCACAACTCTTTTCTCATTTCCAC |
| m-ICAM | GCCTTGGTAGAGGTGACTGAG | GACCGGAGCTGAAAAGTTGTA |
| m-RPL13A | GGATCCCTCCACCCTATGACA | CTGGTACTTCCACCCGACCTC |
| m-VCAM | CCTTGTGGAGGGATGTACAGA | TGCCGAGCTAAATTACACATTG |
