# Supplementary material for: Liver X Receptor Alpha Is Important in Maintaining Blood-Brain Barrier Function
Source: Front Immunol. 2019 Jul 31;10:1811. doi: 10.3389/fimmu.2019.01811 (PMC6685401; doi:10.3389/fimmu.2019.01811)
Supplement: Supplementary file 3 [file Table_3.DOCX]

***Table S2.*** Primary antibodies used for IHC

| **Primary antibody** | **Labeled** | **dilution** | **Species raised in** | **Source** |
| --- | --- | --- | --- | --- |
| Claudin-5 |  | 1:50 | Rabbit | Invitrogen |
| VCAM-1 (MK2.7) | Atto488 | 1:50 | Rat | Amsterdam UMC, VU |
| Rhodamine-lectin |  | 1:50 |  | Vector |
| CD3 |  | 1:150 | Rat | Bio-Rad, Hertfordshire, UK |
| F4/80 |  | 1:100 | Rat | Bio-Rad |
