# Supplementary figures and images for: Liver X Receptor Alpha Is Important in Maintaining Blood-Brain Barrier Function
Source: Front Immunol. 2019 Jul 31;10:1811. doi: 10.3389/fimmu.2019.01811 (PMC6685401; doi:10.3389/fimmu.2019.01811)

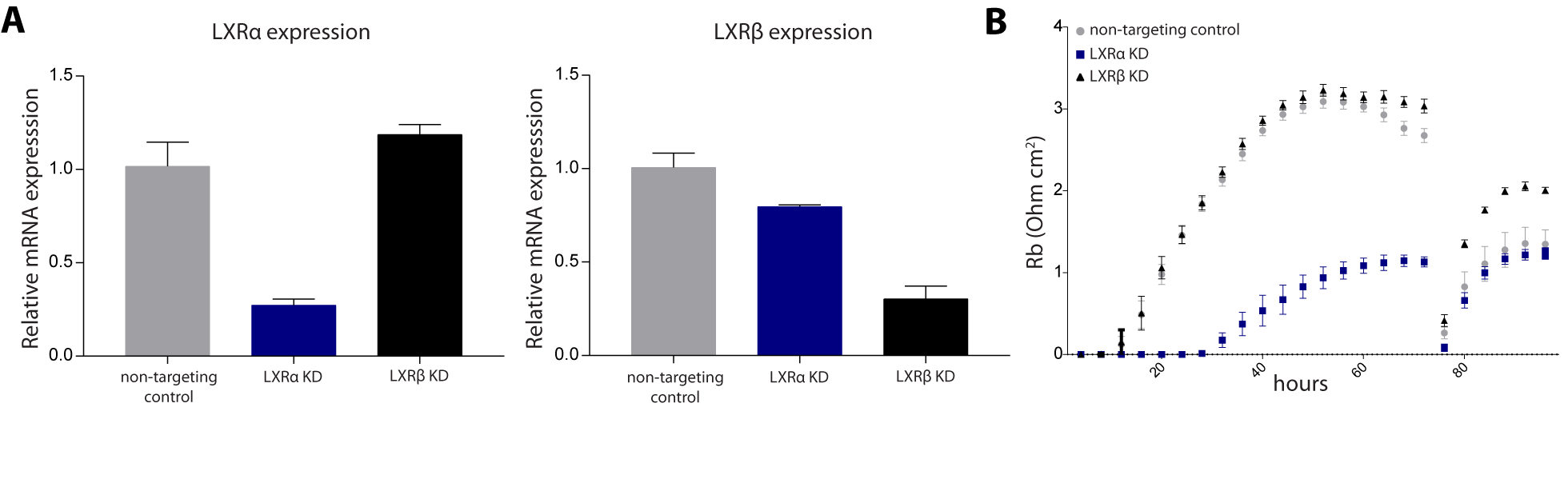

Supplement: Supplementary file 4 [file Image_1.TIF]

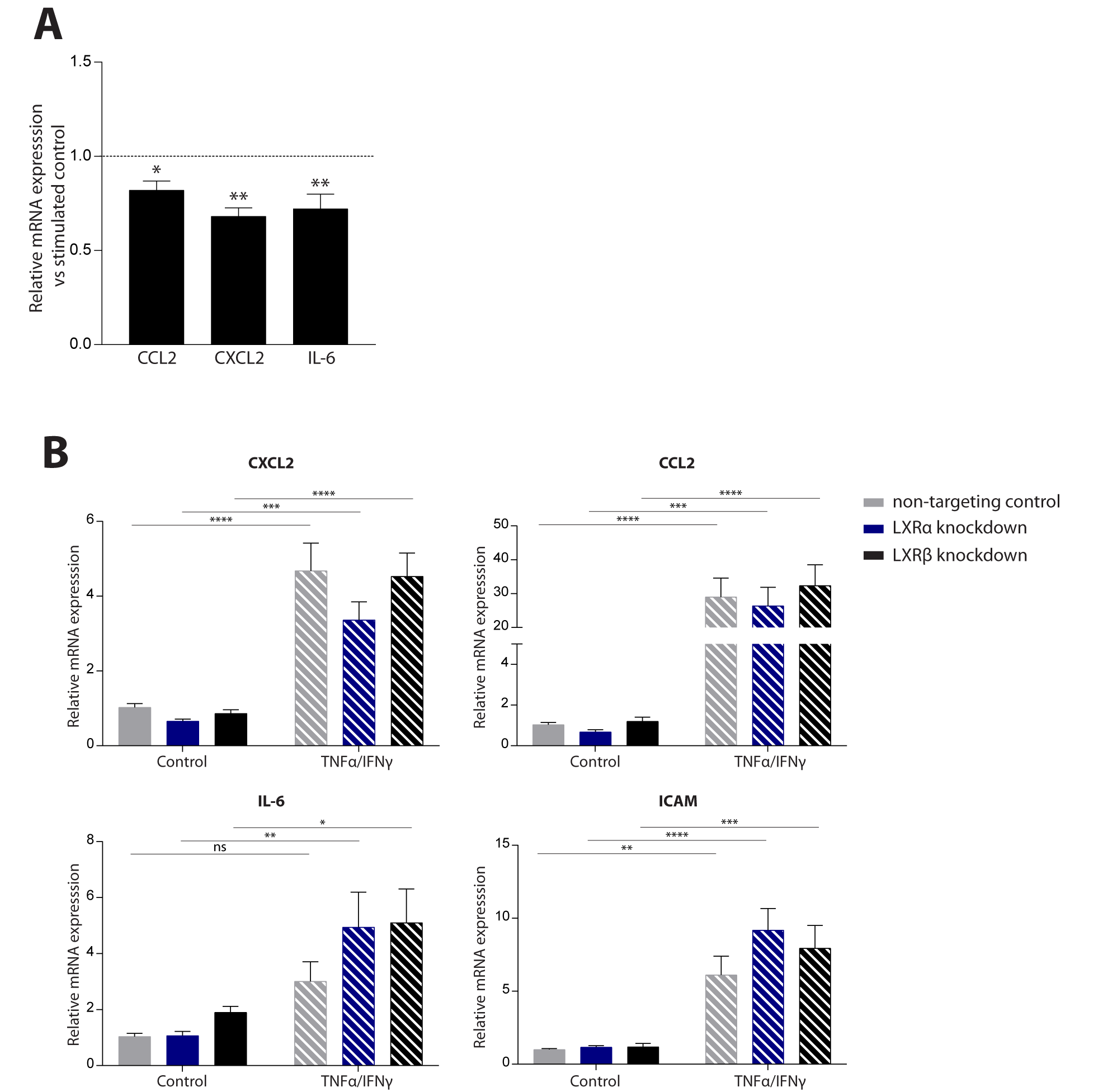

Supplement: Supplementary file 5 [file Image_2.TIF]

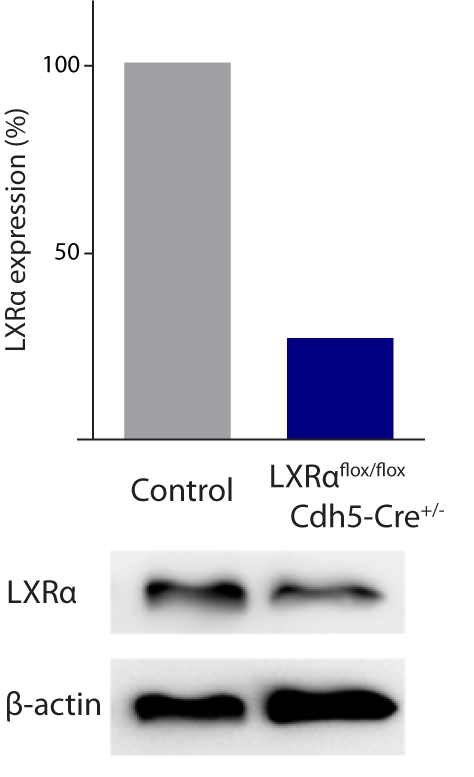

Supplement: Supplementary file 6 [file Image_3.TIF]
